# Dnj1 Promotes Virulence in *Cryptococcus neoformans* by Maintaining Robust Endoplasmic Reticulum Homeostasis Under Temperature Stress

**DOI:** 10.3389/fmicb.2021.727039

**Published:** 2021-09-10

**Authors:** Linda C. Horianopoulos, Christopher W. J. Lee, Guanggan Hu, Mélissa Caza, James W. Kronstad

**Affiliations:** Michael Smith Laboratories, University of British Columbia, Vancouver, BC, Canada

**Keywords:** co-chaperones, JDPs, endoplasmic reticulum, proteostasis, fungal pathogenesis, heat shock

## Abstract

The capacity of opportunistic fungal pathogens such as *Cryptococcus neoformans* to cause disease is dependent on their ability to overcome an onslaught of stresses including elevated temperature under mammalian host conditions. Protein chaperones and co-chaperones play key roles in thermotolerance. In this study, we characterized the role of the endoplasmic reticulum (ER) J-domain containing co-chaperone, Dnj1, in the virulence of *C. neoformans*. A strain expressing a Dnj1-GFP fusion protein was used to confirm localization to the ER, and a *dnj1∆* deletion mutant was shown to be hypersensitive to the ER stress caused by tunicamycin (TM) or 4μ8C. Dnj1 and another ER chaperone, calnexin were found to coordinately maintain ER homeostasis and contribute to maintenance of cell wall architecture. Dnj1 also contributed to thermotolerance and increased in abundance at elevated temperatures representative of febrile patients (e.g., 39°C) thus highlighting its role as a temperature-responsive J domain protein. The elaboration of virulence factors such as the polysaccharide capsule and extracellular urease activity were also markedly impaired in the *dnj1∆* mutant when induced at human body temperature (i.e., 37°C). These virulence factors are immunomodulatory and, indeed, infection with the *dnj1∆* mutant revealed impaired induction of the cytokines IL-6, IL-10, and MCP-1 in the lungs of mice compared to infection with wild type or complemented strains. The *dnj1∆* mutant also had attenuated virulence in an intranasal murine model of cryptococcosis. Altogether, our data indicate that Dnj1 is crucial for survival and virulence factor production at elevated temperatures. The characterization of this co-chaperone also highlights the importance of maintaining homeostasis in the ER for the pathogenesis of *C. neoformans*.

## Introduction

Opportunistic fungal pathogens which occupy an environmental niche encounter several stresses when they gain access to a host. In *Cryptococcus neoformans*, the temperature upshift encountered upon inhalation by an endothermic host or vector is a major stress. Therefore, proteins capable of mitigating stresses and maintaining protein folding capacity, such as the heat shock proteins, may be required for pathogenesis. Previously, heat shock proteins such as Hsp70 and Hsp90 were shown to be required for the virulence of fungal pathogens and have been proposed as potential antifungal therapeutic targets (Cowen et al., 2009; Brown et al., 2010; Sun et al., 2010; Nagao et al., 2012; Cowen, 2013; Cordeiro et al., 2016; Tiwari and Shankar, 2018; Weissman et al., 2020). Along with these major classes of chaperones, the co-chaperones also play important roles in virulence and the elaboration of virulence-related traits such as dimorphic switching, effector secretion, and polysaccharide capsule formation (Lim et al., 2010; Lo Presti et al., 2016; Xie et al., 2017; Horianopoulos et al., 2020; Jurick et al., 2020; Son et al., 2020).

Fungi undergo drastic changes in morphology and metabolism throughout their life cycles. This is particularly true of the opportunistic fungal pathogens which must adapt to host conditions to proliferate. Throughout these changes, the demand for protein production and secretion fluctuates and since many proteins are folded, modified, and assembled in the endoplasmic reticulum (ER), it is crucial that fungi have mechanisms to maintain ER homeostasis and prevent the accumulation of misfolded proteins (Krishnan and Askew, 2014). Achieving this homeostasis requires the coordination of many molecular chaperones in the ER lumen and membrane to ensure that nascent proteins are correctly processed (Brodsky and Skach, 2011). For many proteins synthesized in the ER, processing includes post translational modifications such as glycosylation or packing of transmembrane regions into the membrane (Brodsky and Skach, 2011). If ER homeostasis is not maintained, misfolded proteins accumulate, become toxic, and apoptosis may ensue (Brodsky and Skach, 2011; Delic et al., 2012).

Among fungal pathogens, there is still much to be learned about ER chaperones, although, there are several examples of their importance to virulence, particularly in the context of effector secretion in the plant pathogens. For example, the homolog of the ER Hsp70 (Kar2) nucleotide exchange factor Lhs1 is important for effector translocation in *Magnaporthe oryzae* (Yi et al., 2009) as well as conidiation and virulence in *Fusarium pseudograminearum* (Chen et al., 2019). Additionally, an ER J domain co-chaperone, Dnj1, contributes to effector secretion and virulence in the maize pathogen *Ustilago maydis* (Lo Presti et al., 2016). A distinct type of ER chaperone, protein disulfide isomerase 1 (Pdi1), is also required for virulence and proper effector folding and secretion in *U. maydis* (Marín-Menguiano et al., 2019). ER chaperones also play roles in the virulence of several human fungal pathogens. For example, the ER Hsp70 protein, Kar2, is essential in *Candida albicans* and its role in translocation of secretory proteins suggests a contribution to virulence beyond its essential functions (Morrow et al., 2011). The lectin chaperone, calnexin, also promotes thermotolerance and growth in the presence of ER stress in *Aspergillus fumigatus*; however, it does not contribute to virulence in immunosuppressed mouse models of infection (Powers-Fletcher et al., 2011). Genes encoding ER chaperones are upregulated under stress conditions related to virulence including thermotolerance in *C. neoformans* (Yang et al., 2017). Although a thorough investigation of ER chaperones is limited by the essentiality of some of these proteins such as Kar2 (Morrow et al., 2011; Jung et al., 2013), their upregulation upon temperature upshift suggests they may be important to pathogenesis. Therefore, additional studies on ER chaperones and ER homeostasis in *C. neoformans* are warranted.

While thermotolerance is necessary for survival within a mammalian host, the pathogenesis and dissemination of *C. neoformans* is aided by the secretion of many extracellular factors. In particular, one of the major virulence factors, the capsule, requires the secretion of large amounts of capsular polysaccharide, as well as mannoproteins and capsule modifying enzymes (Doering, 2009; Casadevall et al., 2019). There are several other secreted proteins which contribute to virulence including urease and a metalloprotease, which both promote dissemination into the central nervous system (Cox et al., 2000; Olszewski et al., 2004; Vu et al., 2014). Other exported factors include the extracellular vesicles which are produced by *C. neoformans*. These extracellular vesicles contain virulence-associated proteins and modulate the immune response to *C. neoformans* (Rodrigues et al., 2008; Oliveira et al., 2010). In addition to these secreted factors, there are also cell surface exposed proteins which are folded and modified in the ER. In particular, there are several cell wall-associated proteins in *C. neoformans*, which are required for full virulence including laccase and phospholipase B1 (Siafakas et al., 2007; Panepinto et al., 2009). Due to the requirement of these secreted and cell wall-associated proteins for *C. neoformans* virulence, we expect that ER chaperones will be necessary to maintain ER homeostasis and accommodate the flux in demand for protein folding and secretion during mammalian infection.

In this study, we characterized the role of the ER J domain and tetratricopeptide repeat (TPR) containing co-chaperone Dnj1. The *dnj1∆* deletion mutant displayed a thermotolerance defect as well as a capsule defect at elevated temperatures. Furthermore, the *dnj1∆* mutant was hypersensitive to ER stress and to the azole class of antifungal drugs. The cell wall was thicker in the *dnj1∆* deletion strain, and agents that stabilized the cell wall restored growth of the mutant at elevated temperature. Dnj1 was also important for the extracellular activity of urease at human body temperature. Ultimately, the *dnj1∆* deletion mutant had attenuated virulence in an inhalation mouse model of cryptococcosis compared to the wild type strain, and this was attributed to decreased proliferation based on fewer fungal cells observed in infected lungs and a reduced inflammatory response elicited during the early phase of infection. Overall, we propose that Dnj1 is a co-chaperone which supports ER function and virulence of the human fungal pathogen *C. neoformans*.

## Materials and Methods

### Strains and Media

*Cryptococcus neoformans* var. *grubii* strain H99 (serotype A) was used as the background for mutant construction and as the wild type strain in all experiments. All strains used in this study were routinely maintained on YPD medium (1% yeast extract, 2% peptone, 2% dextrose; BD Difco, Franklin Lakes, NJ, United States). All engineered strains generated for this study including deletion mutants and strains expressing C-terminally tagged fusion proteins (Supplementary Table S1) were produced by biolistic transformation of linear constructs that were prepared using three-step overlap PCR as previously described (Davidson et al., 2002), and using the primers listed in Supplementary Table S2. All chemicals were obtained from Sigma-Aldrich (St. Louis, MO, United States) unless otherwise specified.

### Phylogenetic Analyses

The full length orthologous amino acid sequences to *C. neoformans* Dnj1 (CNAG_01347) were retrieved from UniProt.^1^ These sequences were from *Homo sapiens* (DnaJC3), *U. maydis* (Dnj1; um05173), *Neurospora crassa* (DNAJ protein; NCU02424), *C. albicans* (Jem1; C2_08790W_A), and *Saccharomyces cerevisiae* (Jem1; YJL073W). The sequences were aligned using the ClustalW algorithm in MEGA X (Kumar et al., 2018). A Maximum Likelihood tree was produced using the Le Gascuel amino acid replacement matrix and 500 bootstrap trees to assess the robustness of the resultant tree (Le and Gascuel, 2008). The presence and location of TPR was predicted using TPRpred^2^ (Karpenahalli et al., 2007). Predicted TPR regions with a value of *p* <0.01 were indicated on a schematic and the overall probability that the sequence was a TPR protein was determined.

The amino acid sequences of the J domains from these proteins were retrieved from UniProt and independently aligned using the ClustalW algorithm in MEGA X (Kumar et al., 2018). The alignment was visualized using pyBoxshade v 1.1.2 (GitHub – mdbaron42/pyBoxshade, n.d.).^3^

### Dnj1 Localization

A strain expressing a C-terminal fusion of GFP to Dnj1 was constructed in the background of the *dnj1Δ* mutant (Supplementary Table S1). The Dnj1-GFP expressing cells were grown overnight in YNB+0.5% glucose and stained for 30min at room temperature with 200nM ER-Tracker™ (Invitrogen, Carlsbad, CA) in Hank’s balanced salt solution with calcium and magnesium or 5μg/ml DAPI in phosphate buffered saline (PBS). Cells were imaged using a Zeiss Plan-Apochromat 100x/1.46 oil lens on a Zeiss Axioplan 2 microscope. Images were obtained using an ORCA-Flash4.0 LT digital CMOS camera (Hamamatsu, Hamamatsu City, Japan). All fluorescent images were processed using Zen 3.0 software (Zeiss, Oberkochen, Germany).

### Growth Assays

All growth assays performed in liquid media were conducted in 96-well plates in a final volume of 200μl inoculated with 2×10^4^ cells. YNB with amino acids and 0.5% dextrose (BD Difco) was used as the base media and as a control to assay the susceptibility of strains to 150ng/ml tunicamycin (TM) and 20μM 4μ8C. Plates were incubated at 30°C with shaking at 140rpm and read using a Tecan M200 Infinite plate reader (Tecan, Männedorf, Switzerland). Growth curves and SD were generated in GraphPad Prism 7 (GraphPad Software, San Diego, CA, United States).

Hypersensitivity to ER stressors, azole drugs, cell wall stress, and temperature stress was assessed on solid media using 10-fold serial dilutions of cells spotted onto YPD agar supplemented with 150ng/ml tunicamycin, 10mM dithiothreitol (DTT), 50ng/ml miconazole (MCZ), 10μg/ml fluconazole (FLZ), 1M sorbitol, 1.5M NaCl, or 0.5mg/ml caffeine. Cells were grown overnight in YPD, washed in sterile water, diluted to 20,000 cells per μl, 10-fold serially diluted, and spotted on solid media. Spot assays were also performed to evaluate melanin formation on chemically defined media containing 0.1% L-asparagine, 0.1% dextrose, 3mg/ml KH_2_PO_4_, 0.25mg/ml MgSO_4_∙7H_2_O, 1μg/ml thiamine, 5ng/ml biotin, and 0.2mg/ml L-3,4-dihydroxyphenylalanine (L-DOPA). Plates were incubated at 30, 37, or 39°C as indicated for 2–5days before being scanned to assess differences in growth between strains.

### RNA Extraction and RT-qPCR

Wild type *C. neoformans* was grown overnight in 5ml YPD cultures, diluted 1 in 10 in fresh YPD in a final volume of 25ml and grown for 6h in 250ml Erlenmeyer flasks. The expression of *DNJ1* was studied under ER stress and elevated temperature conditions by collecting cells by centrifugation and resuspending them in media supplemented with 250ng/ml tunicamycin or in pre-warmed to 37°C, with further incubation for 1h. Cells were then collected by centrifugation, washed once in ice cold sterile water, flash frozen in liquid nitrogen and stored at −80°C. Cell pellets were resuspended in buffer RLT (Qiagen, Hilden, Germany) and lysed by bead beating twice for 1min increments with 1min on ice between bead beating. Total RNA was extracted using a RNeasy kit (Qiagen) following the manufacturer’s instructions for yeast with mechanical disruption. Total RNA was treated with Turbo DNase (Ambion, Austin, TX, United States) according to the manufacturer’s instructions and cDNA was synthesized using the Verso cDNA reverse transcription kit using oligo(dT) to select for polyadenylated mRNA (ThermoFisher). RT-qPCR was performed using Green-2-Go qPCR Mastermix Low ROX (Bio Basic, Amherst, NY, United States) with the primers listed in Supplementary Table S2. The reactions were performed on an Applied Biosystems 7,500 Fast real-time PCR system. Relative gene expression was determined using the 2^-∆∆CT^ method (Livak and Schmittgen, 2001) and normalized to *ACT1* and *GAPDH* expression. Statistically significant differences were evaluated using unpaired *t*-tests (GraphPad).

### Protein Extraction and Immunoblotting

Strains expressing a C-terminally HA-tagged Dnj1 fusion protein were grown overnight in 5ml of YPD, transferred to 45ml of fresh YPD and grown at 30°C with shaking at 200rpm for 6h. Cells were collected by centrifugation and resuspended in pre-heated YPD (at 37 or 39°C) or YPD supplemented with 250ng/ml tunicamycin, and incubated for an additional hour. Cell pellets were collected by centrifugation, flash frozen in liquid nitrogen, and extracted as previously described (Horianopoulos et al., 2020). Protein concentration was measured using a Pierce BCA Protein Assay kit following the manufacturer’s instructions (ThermoFisher). For immunoblots to determine changes in protein abundance, 40μg of total protein was subjected to electrophoresis in each well of a 10% SDS-PAGE gel. Protein was transferred to a PVDF membrane in a wet transfer for 3h at 70V. Membranes were probed with 1:10000 anti-HA monoclonal antibody (2-2.2.14, ThermoFisher) and THE™ beta Actin antibody (A00702, GenScript, Piscataway, NJ, United States) followed by 1:5,000 anti-mouse HRP (#1706516, Bio-Rad, Hercules, CA, United States). Changes in protein abundance relative to actin were measured using ImageJ (Schneider et al., 2012). Membranes were also stained with Ponceau S to ensure equal loading.

### Assessment of Capsule Formation

Low iron capsule inducing media (CIM) was used to induce capsule formation as previously described (Lian et al., 2004). Briefly, cells were grown overnight in YPD, washed in sterile low iron water, and 10^6^ cells/ml were inoculated in CIM. Cells were imaged after 48h of growth at 30 or 37°C (stained 1:1 with India ink). The capsule thickness and cell diameter were measured for 50 cells from each strain using ImageJ (Schneider et al., 2012), and the differences in capsule size between strains were evaluated using an ANOVA with Tukey’s multiple comparisons in GraphPad (GraphPad).

### Extracellular Urea Hydrolysis Assay

The hydrolysis of extracellular urea by secreted urease was determined using minimal media (MM) with the addition of 2% urea and 0.0012% phenol red as a pH indicator prepared as previously described (Choi et al., 2012) with the modification of using liquid media to allow quantification of the pH change in the media using a plate reader (Tecan). Briefly, cells were grown overnight in YPD, washed three times in sterile water, and inoculated at 10^6^ cell/ml in 200μl of MM+urea+phenol red in a 96-well plate. The secretion of urease was quantified by measuring the absorbance of the spent media at 570nm after 48h of growth at either 30 or 37°C as indicated. Statistical significance was determined using ANOVA’s with Tukey’s multiple comparisons in GraphPad Prism 7 (GraphPad Software).

### Flow Cytometry

Cells were grown for 16h in YNB+0.5% dextrose at 30 or 37°C and diluted to an OD_600nm_ of 1 for staining of surface exposed cell wall components. Chitin was stained with 100μg/ml calcofluor white (CFW) in PBS and chitosan was stained with 250μg/ml eosin Y in McIlvaine’s buffer pH 6.0 for 15min at room temperature in the dark as previously described (Santiago-Tirado et al., 2015). All flow cytometry data were collected on an Attune Nxt Flow Cytometer (Invitrogen). Eosin Y was detected with the BL1 filter and CFW was detected with the VL1 filter. Flow cytometry data were analyzed using FlowJo v10 software (FlowJo, LLC, Ashland, OR, United States) and statistical significance was evaluated by performing ANOVA’s with Tukey’s multiple comparisons in GraphPad Prism 7 (GraphPad Software).

### Transmission Electron Microscopy

Cells were grown overnight in YNB at either 30 or 37°C and normalized to OD_600nm_ of 1. Cells were washed three times in PBS and fixed in 4% formaldehyde and 2.5% glutaraldehyde in 0.1M sodium cacodylate pH 6.9. After fixation, cells were separated in 3% low temperature gelling agarose and post fixed in 2% OsO_4_ for 1h. The cells were washed three times with ddH_2_O and dehydrated through sequential washes with a graded concentration series of ethanol into 100% ethanol. After dehydration, cells were embedded in Spurr’s resin and 70nm sections were cut using a Leica Ultramicrotome UCT. Sections were stained with 2% uranyl acetate for 20min, followed by 2% lead citrate for 10min. Images were taken on a Hitachi 7,600 transmission electron microscope operating at 80kV and images were acquired with an AMT XR51 camera. For each cell imaged, the cell wall thickness was measured at four points using ImageJ (Schneider et al., 2012) and the average cell wall thickness was determined. Statistical significance was determined by performing an ANOVA with Tukey’s multiple comparisons in GraphPad Prism 7 (GraphPad Software).

### Virulence Assays

Inocula were prepared by growing WT, *dnj1Δ* mutant, and *dnj1Δ*::Dnj1HA cells in YPD overnight at 30°C, washing three times in sterile PBS (Gibco, Waltham, MA, United States), and resuspending at 4.0×10^6^ cells/ml in PBS. Ten female BALB/c mice aged 4–6weeks old (Charles River Laboratories, ON, Canada) were anesthetized intraperitoneally with 80mg/kg ketamine and 5.5mg/kg xylazine and inoculated with each strain by intranasal instillation with 50μl of cell suspension (inoculum of 2×10^5^ cells per mouse). Infected mice were monitored daily post-inoculation and upon displaying signs of morbidity, the mice were euthanized by carbon dioxide anoxia. For the determination of fungal burdens in organs at endpoint, cardiac blood was retrieved and organs were excised, weighed, and homogenized in two volumes of PBS using a MixerMill MM400 (Retsch, Haan, Germany). Serial dilutions of the homogenates were plated on YPD agar plates containing 50μg/ml chloramphenicol, and colony forming units (CFU’s) were counted after incubation for 48h at 30°C. Significance in survival assays was determined using log-rank tests and significance in fungal burden was determined using Mann-Whitney U tests in GraphPad Prism 7 (GraphPad Software).

Lungs collected for histology were fixed overnight in 10% formalin. Samples were embedded in paraffin wax, sectioned, and stained with either hematoxylin and eosin or mucicarmine by Wax-it Histology Services Inc. (Vancouver, Canada). Sections were visualized using a Zeiss Axioskop2 microscope equipped with a Zeiss AxioCam HRc camera (Zeiss).

### Phagocytosis Assay

Phagocytosis assays were performed using the J774.A1 murine macrophage cell line (ATCC, Manassas, Virginia). J774.A1 cells were grown in Dulbecco’s Modified Eagle Medium (DMEM, Gibco) supplemented with 10% Fetal Bovine Serum, 100units/ml penicillin, 100μg/ml streptomycin, and 2mM L-glutamine at 37°C with 5% CO_2_. J774.A1 cells used for the experiments were kept between passages 5 and 20. Macrophages were seeded at a density of 200,000 cells/ml 24h prior to infection in eight-well chamber slides with removable wells. On the day of infection, overnight cultures of *C. neoformans* grown in YPD media at 30°C were washed three times with PBS and opsonized with 10μg/ml of 18b7 monoclonal antibody in serum-free DMEM for 1h at room temperature. Simultaneously, macrophages were activated with 150ng/ml of phorbol 12-myristate 13-acetate in serum-free DMEM for 1h prior to infection. Macrophages were then incubated with the opsonized cryptococcus for 2h at a multiplicity of infection 1:10 (macrophage:yeast) at 37°C with 5% CO_2_. Immediately after incubation, live images were taken using eight-chambered removable well slides and at least 200 macrophages were counted per strain for each experiment. Three independent experiments were conducted and significance was determined using a one-way ANOVA with Tukey’s multiple comparisons in GraphPad Prism 7 (GraphPad Software).

### Lung Cytokine Measurements

Inocula were prepared as described for the virulence assay and seven female BALB/c mice aged 4–6weeks old (Charles River Laboratories) were inoculated with each strain by intranasal instillation with 50μl of cell suspension (inoculum of 2×10^5^ cells per mouse) or mock inoculated with 50μl PBS. Lung tissue was collected from mice 6days post inoculation and homogenized at 25cycles/s for 5min in 1ml of PBS containing complete EDTA-free protease inhibitor cocktail (Roche, Basel, Switzerland) using a mixer mill (Retsch). The insoluble tissue was removed from the supernatant through centrifugation at 4500×*g* for 15min at 4°C and the supernatant was stored at −80°C. Cytokines were captured and quantified using the BD cytometric bead array mouse inflammation kit following the manufacturer’s instructions (BD Biosciences, Franklin Lakes, NJ, United States). The beads were analyzed on an Attune Nxt Flow Cytometer (Invitrogen) using the YL-1 and RL-1 filters to detect PE and APC, respectively. The data was analyzed using FlowJov10 software (FlowJo, LLC, Ashland, OR, United States) and statistical significance was evaluated by performing ANOVA’s with Tukey’s multiple comparisons in GraphPad Prism 7 (GraphPad Software).

All experiments with mice were conducted in accordance with the guidelines of the Canadian Council on Animal Care and approved by the University of British Columbia’s Committee on Animal Care (protocol A17-0117).

## Results

### Dnj1 Is a Tetratricopeptide Repeat and J Domain Containing Co-chaperone

**Figure 1:**
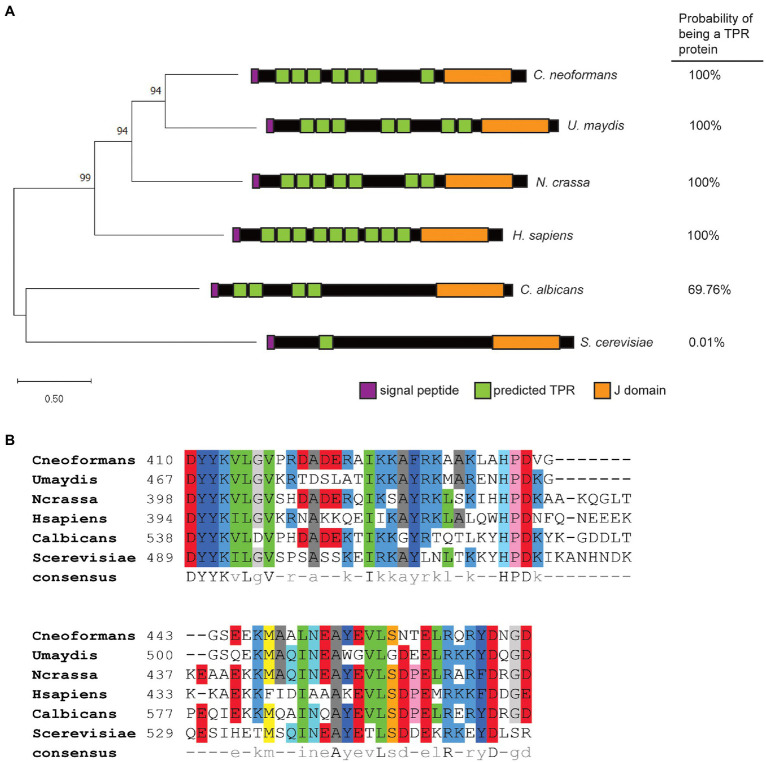
Dnj1 is a tetratricopeptide repeat and J domain-containing protein distinct from the Jem1 proteins in the Saccharomycotina. **(A)** A Maximum Likelihood tree constructed based on a ClustalW alignment of amino acid sequences of Dnj1 and putative orthologs retrieved from UniProt. The Le Gascuel amino acid replacement matrix was used to build the tree and the bootstrap values of *p* from 500 bootstrap trees are indicated. A schematic of each protein is shown indicating the predicted TPRs with values of *p*<0.01 predicted by TPRpred and the overall probability that each protein is a tetratricopeptide repeat (TPR) is specified. **(B)** An amino acid alignment of the J domains for the putative ortholog from each indicated species. The consensus amino acid sequence determined using pyBoxshade is indicated at the bottom of the alignment.

Dnj1 (CNAG_01347) is orthologous to the recently characterized Dnj1 in *U. maydis* (um05173; Lo Presti et al., 2016). Both proteins contain seven putative TPRs predicted in TPRpred (Karpenahalli et al., 2007) and have a strong overall prediction of being TPR proteins (Figure 1A). Similarly, *N. crassa* has a conserved TPR and J domain containing co-chaperone suggesting that Dnj1 is not a basidiomycete specific protein (Figure 1A). Previously, Dnj1 in *C. neoformans* was described as a putative ortholog of Jem1/Kar8 from *S. cerevisiae*, although, it had no influence on karyogamy and mating, thus suggesting that it may diverge in function from Jem1 (Lee and Heitman, 2012). In support of this idea, we found that the protein sequence of Dnj1 is divergent from the Jem1 proteins in the Saccharomycotina using *C. albicans* and *S. cerevisiae* Jem1 as representative sequences (Figure 1A). Both Jem1 proteins had relatively few predicted TPR regions and lower overall TPR protein prediction scores, further indicating their divergence from Dnj1. Indeed, Dnj1 shares greater amino acid sequence similarity to human Erdj6 (DnajC3), another TPR-containing J domain co-chaperone that functions in the ER (Rutkowski et al., 2007), than it does to the Jem1 proteins (Figure 1A).

Although, the major divergence between Dnj1 and the Jem1 proteins appeared to be in the number of TPRs, recent work highlighted that functional differences in J domain proteins (JDPs) can be afforded by divergence in the amino acid sequence of the J domain itself (Piette et al., 2021). Therefore, we aligned the amino acid sequences of the J domains from these proteins (Figure 1B). Interestingly, one of the major differences in the J domain sequence was a segment after the conserved HPD motif, which was absent in the proteins from both of the basidiomycetes (Figure 1B). Altogether, the similarities between Dnj1 in *C. neoformans* and *U. maydis* suggest that they may participate in similar roles.

### Dnj1 Is Localized to and Supports the Function of the ER in *C. neoformans*

**Figure 2:**
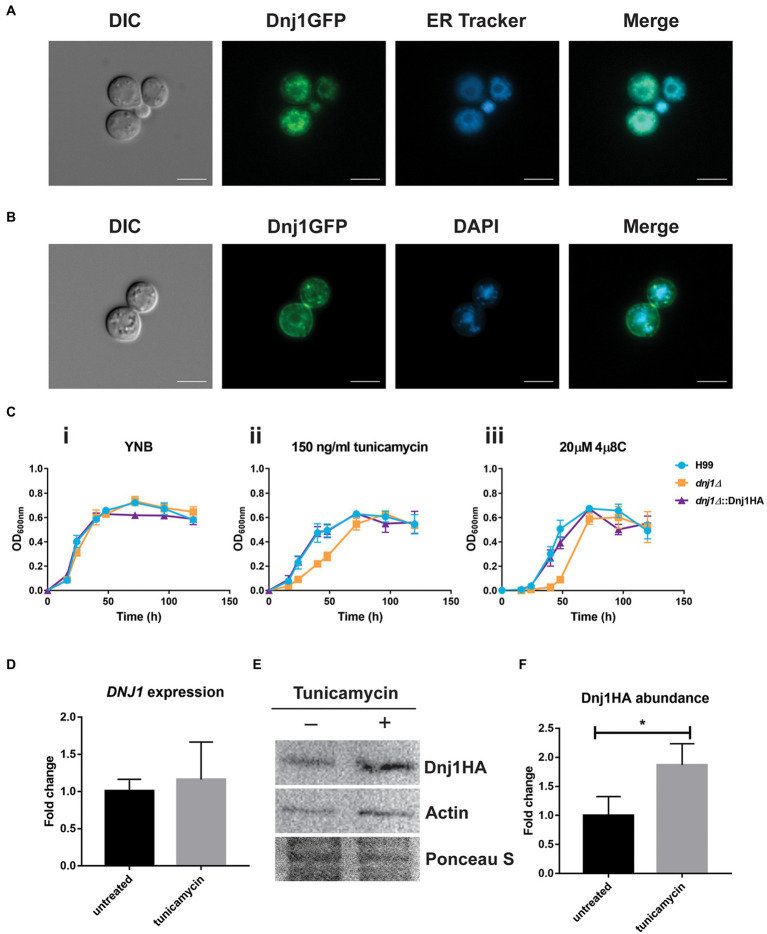
Dnj1 is localized to the endoplasmic reticulum (ER) and is responsive to ER perturbing agents. **(A)** A strain expressing a C-terminally GFP tagged Dnj1 protein was stained with ER-Tracker™ to assess co-localization. **(B)** To confirm that the ring of fluorescence consistently visualized in the Dnj1-GFP expressing strains was perinuclear ER, the DNA in nuclei was stained with DAPI. Bar=5μm. All microscopy images are representative of at least 20 images. **(C)** Growth curves in liquid YNB+0.5% dextrose supplemented with ER inhibitors revealed hypersensitivity of the deletion mutant *dnj1∆* to 150ng/ml tunicamycin (TM) and to 20μM 4μ8C compared to the wild type (H99) or complemented strain (*dnj1∆*::Dnj1HA). The error bars represent the SD of three biological replicates. **(D)** RT-qPCR demonstrating the expression of *DNJ1* in response to treatment with 250ng/ml tunicamycin for 1h. **(E)** Detection of the abundance of Dnj1HA in response to tunicamycin treatment by immunoblot analysis. The blot was probed for actin as well to allow relative quantification and stained with Ponceau S to ensure equal protein loading. This blot is representative of three blots which were quantified in **(F)**. In both **(D)** and **(F)**, bar heights indicate the mean of three replicated and error bars indicate the SD. Statistical significance was determined using unpaired *t*-tests (^*^p<0.05).

Dnj1 has a predicted signal peptide and its ortholog in *U. maydis* was shown to localize to the ER, where it was hypothesized to assist in response to ER stress and in the correct folding of proteins destined for secretion (Lo Presti et al., 2016). We therefore generated a strain expressing Dnj1 with a C-terminal GFP tag (Dnj1-GFP) and stained these cells with ER Tracker™ to reveal that Dnj1 co-localized with the ER (Figure 2A). We consistently observed rings of GFP signal in the Dnj1-GFP strain which we hypothesized to be perinuclear ER. This idea was confirmed through co-staining with DAPI to determine that the rings of Dnj1-GFP indeed surrounded nuclei (Figure 2B). Taken together, these data indicate that Dnj1 is localized to the ER in *C. neoformans*, including the perinuclear ER.

To interrogate the impact of *DNJ1* in growth and virulence, a deletion mutant and a C-terminally HA-tagged complement were generated and confirmed by PCR and Southern blotting (Supplementary Figure S1). When challenged with ER stress, the *dnj1∆* deletion mutant was hypersensitive compared to the wild type (H99) and complemented (*dnj1∆*::Dnj1HA) strains (Figure 2C; Supplementary Figure S2). In liquid growth assays, the *dnj1∆* deletion mutant had a growth defect compared to the wild type and complemented strains when grown in the presence of the N-linked glycosylation inhibitor, tunicamycin, or with the Ire1 RNase inhibitor, 4μ8C (Figure 2C). Importantly, expression of the Dnj1GFP fusion protein also complemented growth in the presence of these ER inhibitors (Supplementary Figure S3). In particular, the mutant had a longer lag phase when grown in the presence of these inhibitors. Growth on solid media revealed that the deletion mutant grew very poorly; however, a few colonies eventually appeared suggesting the emergence of resistance (Supplementary Figure S2); this may possibly explain the observed eventual growth of the mutant to the wild type level in liquid media (Figure 2C). Furthermore, in contrast to the mutant lacking the Dnj1 ortholog in *U. maydis*, a mild sensitivity on dithiothreitol was observed when *C. neoformans* was grown on solid medium at 37°C (Supplementary Figure S2). Although, *DNJ1* was not significantly upregulated at the transcriptional level after treatment with tunicamycin (Figure 2D), Dnj1 with a C-terminal HA tag was more abundant at the protein level after tunicamycin treatment (Figures 2E,F). This result suggests that Dnj1 responds to the ER stress elicited by tunicamycin.

### Dnj1 Cooperates With the ER Chaperone Calnexin to Permit Robust Growth

**Figure 3:**
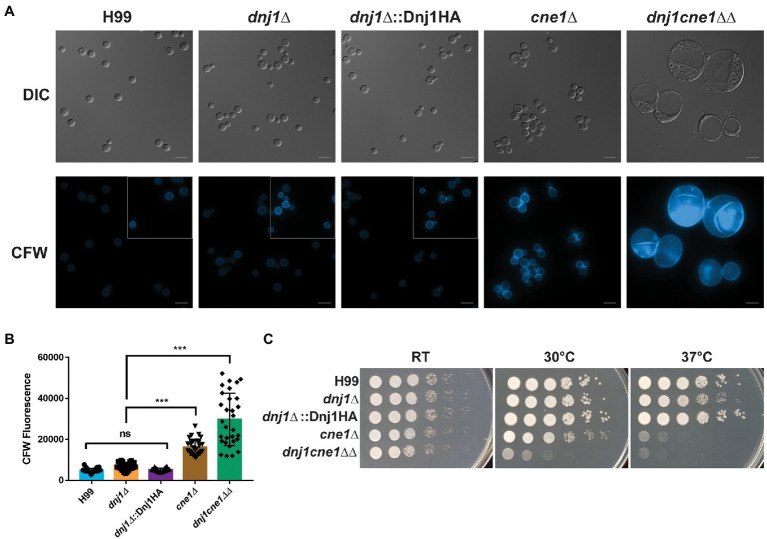
Dnj1 and calnexin are required for robust growth under routine culture conditions. Calnexin (*CNE1*) was deleted in the background of a *dnj1∆* deletion mutant and the resultant double deletion mutant grew poorly under routine culture conditions. **(A)** Microscopy of the wild type (H99), single knockouts (*dnj1∆* and *cne1∆*), Dnj1HA complement, and double knockout (*dnj1cne1∆∆*) grown overnight in YPD under routine conditions (30°C). Cells stained with 100μg/ml of calcofluor white (CFW) are shown to reveal changes in surface exposed chitin in the mutants. The images of all CFW stained cells have consistent brightness and contrast applied to all images to highlight the differences in staining. However, since H99, *dnj1∆*, and *dnj1∆*::Dnj1HA had less CFW staining, the subsections in the gray boxes have enhanced brightness to show the morphology of the cells for these strains. Bar=10μm. **(B)** Quantification of the average fluorescence intensity from CFW per cell for 30 cells was measured for each genetic background. Each dot represents a measured cell, the mean is represented by the bar height and the SD is shown with error bars. Statistical significance was determined using a one-way ANOVA with Tukey’s multiple comparisons (ns, not significant; ^***^p<0.005). **(C)** Spot assays of serially diluted strains on YPD plates incubated at different temperatures (RT, 30 or 37°C) for 2days before being scanned. Microscopy images and spot assays are representative of three independent replicates.

Typically, the ER chaperones coordinate their activity and act together to ensure that client proteins are correctly processed. Since the *dnj1∆* deletion showed hypersusceptibility to the N-glycosylation inhibitor tunicamycin, we constructed a double deletion strain lacking both *DNJ1* and the lectin type chaperone calnexin (*CNE1*; CNAG_02500), which functions to stabilize non-native glycoproteins and retain them in the ER until they are properly folded or targeted for ER associated degradation (ERAD; Williams, 2006). The resultant double deletion mutant (*dnj1cne1∆∆*) grew more poorly than either single mutant under routine growth conditions in rich media (YPD) at 30°C (Figure 3). This growth defect was associated with an abnormal cell morphology of enlarged cells with collapsed cell walls. Furthermore, the cell walls of the double knockout had significantly more chitin as determined by CFW staining (Figures 3A,B).

A genetic interaction between the mutants was suggested by the more severe growth phenotype observed for the double mutant at elevated temperatures. That is, the *cne1∆* single deletion mutant had a slight growth defect at 30°C and marked defect at 37°C, whereas the *dnj1cne1∆∆* deletion strain had poor growth at both 30 and 37°C compared to the wild type and complemented strains (Figure 3B). In addition, the growth defect at 37°C in the double mutant and *cne1∆* single mutant was striking compared to the mild defect seen for the *dnj1∆* single mutant. Robust growth of the double mutant was achieved upon incubation on YPD at room temperature. These results indicate that although each of these proteins contribute to tolerance of elevated temperatures, they are both required to permit a wild type level of growth under routine conditions at 30°C. The implication is that there is considerable dependence on the ER chaperones to maintain folding capacity and homeostasis even under routine laboratory growth conditions. These results are also consistent with an enlarged cell size observed in a *U. maydis dnj1cne1∆∆* (Lo Presti et al., 2016); however, the temperature associated phenotypes may reflect a novel role for these proteins in the facilitating survival of *C. neoformans* in a mammalian host.

### Dnj1 Influences Fungal Specific Drug Targets at Elevated Temperatures: Ergosterol and the Cell Wall

**Figure 4:**
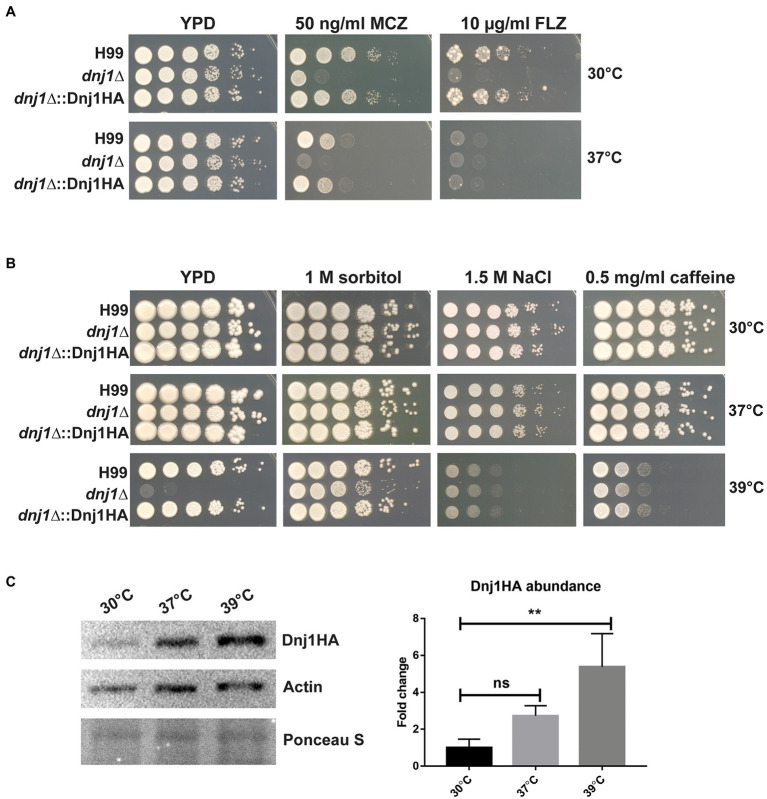
Dnj1 impacts sensitivity to azoles, cell wall stress, and temperature stress. Spot assays of serially diluted *Cryptococcus neoformans* wild type (H99), *dnj1∆* mutant, and complemented (*dnj1∆*::Dnj1HA) strains were performed on YPD supplemented with the indicated stressors and incubated at 30, 37, or 39°C to assess hypersensitivity. **(A)** The sensitivity of the *dnj1∆* mutant to the antifungal azole drugs, fluconazole (FLZ), and miconazole (MCZ), at the indicated concentrations in YPD agar, was assessed at both 30 and 37°C. **(B)** The impact of agents that provoke osmotic and cell wall stress on the growth of the *dnj1∆* mutant was evaluated at 30, 37, and 39°C in YPD supplemented with the indicated concentrations of these stress-inducing agents. For all spot assays, plates were incubated for 2–5days at the indicated temperature before being scanned. The spot assays shown are representative of at least three independent replicates. **(C)** Detection of the abundance of Dnj1HA in response to temperature stress by immunoblot analysis. The blot was also probed for actin to allow relative quantification and Ponceau S stained to ensure equal protein loading. This blot is representative of three experiments that established the relative abundance of the Dnj1HA protein. The bar heights indicate the mean of three replicated and error bars indicate the SD. Statistical significance was determined using a one-way ANOVA with Dunnet’s multiple comparison tests (^**^p<0.01).

Two of the major targets for current antifungal drugs include the membrane lipid ergosterol and the fungal cell wall (Odds et al., 2003). Since sterol biosynthesis and the folding of many glycosylated and GPI-anchored proteins targeted to the cell wall primarily occurs in the ER (Gaynor et al., 1999; Castillon et al., 2009; Hu et al., 2017), we tested the susceptibility of the *dnj1∆* mutant to the azole drugs as well as cell wall stress. The *dnj1∆* mutant was found to be hypersensitive to the azole drugs fluconazole and miconazole at both 30 and 37°C (Figure 4A). The influence of Dnj1 on cell wall integrity was assayed using hyperosmotic conditions (sorbitol), salt stress, and caffeine. Interestingly, the agents that provoked cell wall and osmotic stress did not impair growth of the *dnj1∆*, but rather restored the growth of the mutant at high temperature (39°C; Figure 4B). The mutant was unable to grow at 39°C on YPD, prompting us to evaluate the abundance of Dnj1HA under temperature stress. Dnj1HA was found to increase in abundance after incubation at elevated temperatures, and this increase was statistically significant after incubation at 39°C for 1h (Figure 4C). Together, these results suggest that the expression of Dnj1 is temperature responsive and that an inability to stabilize the cell wall in the *dnj1∆* mutant contributes to the observed temperature sensitivity.

**Figure 5:**
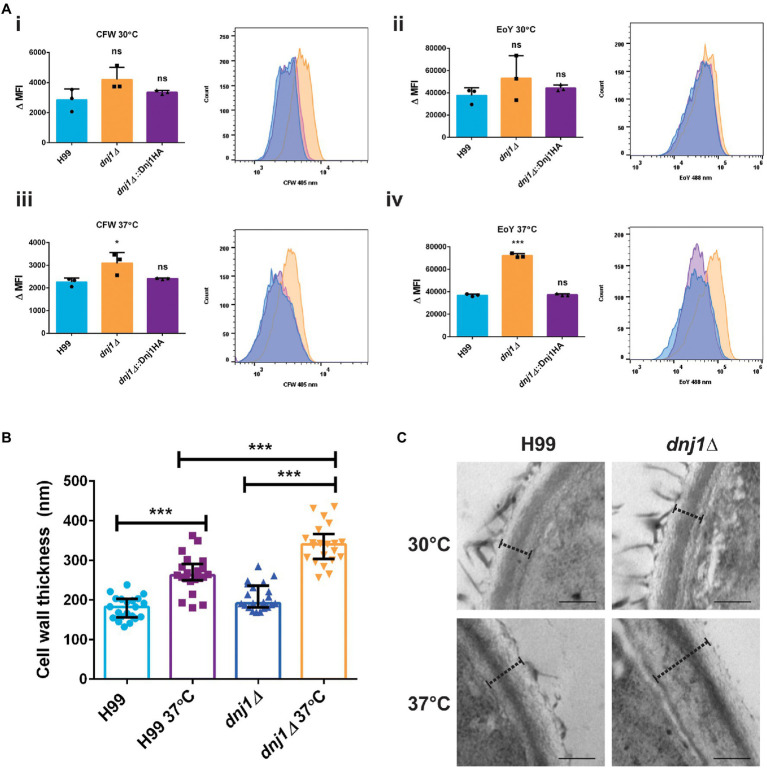
The absence of *DNJ1* alters the cell wall of *C. neoformans*. **(A)** The relative amounts of surface exposed cell wall components in the wild type (H99), *dnj1∆* mutant, and complemented strains (*dnj1∆*::Dnj1HA) were evaluated after growth at 30 or 37°C using CFW to stain chitin and eosin Y (EoY) to stain chitosan. The amount of staining was determined by quantifying mean fluorescence intensity (MFI) measured using flow cytometry. The results of three biological replicates are shown as individual dots, the bars represent the means, and error bars represent SD. Representative histograms of fluorescence intensity are also shown for each condition. **(B)** The cell wall thickness of the wild type and deletion mutant were measured from images obtained using transmission electron microscopy (TEM). For each condition, the cell walls of at least 20 cells were measured as indicated by dashed lines at four distinct points and averaged. The average cell wall thickness per cell is plotted as individual dots and the average and SD of measurements for each group are shown. **(C)** Representative images of the cell walls measured, scale bar=200nm. Statistical significance was determined using one way ANOVA’s with Tukey’s multiple comparisons (ns, non-significant; ^*^p<0.05 and ^***^p<0.005).

Since osmotic and cell wall stress restored the growth of the *dnj1∆* mutant at elevated temperatures, we hypothesized that stabilization of the cell wall or activation of the cell wall integrity pathway may explain this observation. These results prompted further characterization of the impact of Dnj1 on the cell wall particularly at elevated temperatures. Flow cytometry was used to detect fluorescently stained cell wall components and to compare the levels of surface exposed chitin and chitosan between strains. The *dnj1∆* deletion strain was found to have significantly increased surface exposed chitin and chitosan particularly when grown at the human body temperature of 37°C (Figure 5A). This increase as well as the restoration of growth at high temperature by hyperosmotic media (Figure 4B) also prompted the investigation of cell wall thickness upon temperature upshift. Accordingly, transmission electron microscopy (TEM) was used to assess differences in cell wall thickness as well as any changes in ultrastructure of the *dnj1∆* mutant upon growth at human body temperature. Cell wall thickness of the cells visualized using TEM was found to be greater after growth at 37°C compared to 30°C. This thickening of the cell wall occurred in both the wild type and *dnj1∆* strains, although, the cell wall of the *dnj1∆* mutant was significantly thicker than that of the wild type grown at 37°C (Figures 5B,C). Capsular fibers external to the cell wall were also observed; however, relatively low amounts of capsule fibers were observed on the *dnj1∆* mutants grown at 37°C. Otherwise, the ultrastructure was unchanged between strains and growth conditions (Figure 5C).

### Virulence Factor Production in the *dnj1∆* Mutant at Physiologically Relevant Temperatures

**Figure 6:**
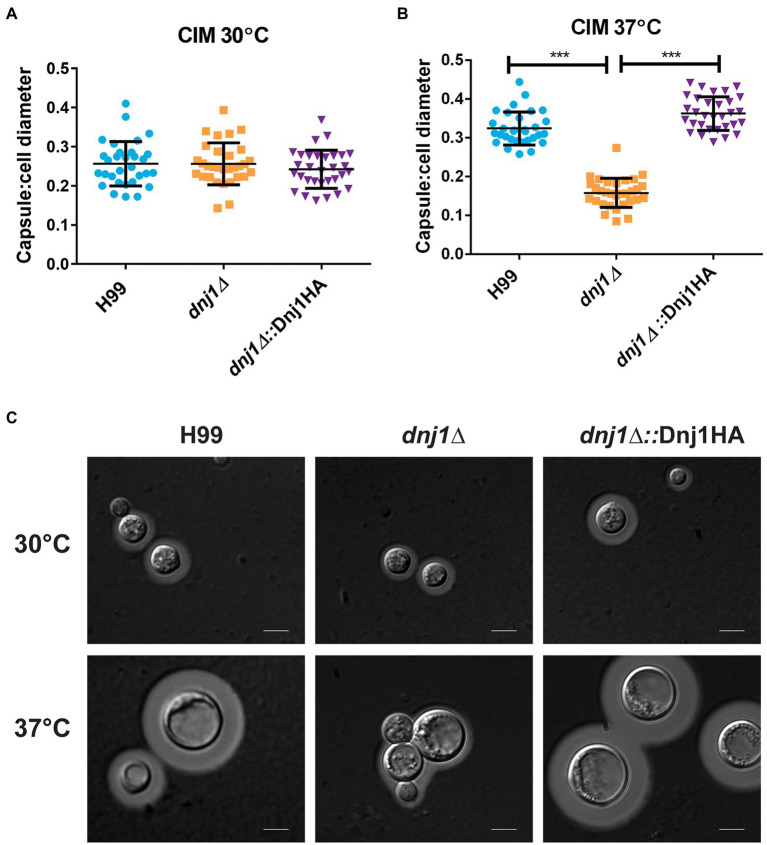
Dnj1 is required for capsule synthesis at human body temperature. Capsule synthesis was induced in low iron capsule inducing media (CIM) at 30 and 37°C for 48h. **(A,B)** The capsule width and cell diameters of 30 cells each from the wild type (H99), deletion mutant (*dnj1∆*), and complement (*dnj1∆*::Dnj1HA) strains were measured. The ratio of capsule thickness to cell diameter for each cell measured is shown as an individual dot. The mean and SD are shown for each group. Statistical significance was determined using a one way ANOVA with Tukey’s multiple comparison tests (^***^p<0.005). **(C)** Representative images are shown of capsule stained with India ink for each strain at the indicated temperatures. Scale bar=5μm.

The elaboration of one of the major virulence factors, the polysaccharide capsule, requires the secretion of polysaccharides, mannoproteins, and extracellular capsule-modifying enzymes (Doering, 2009; Casadevall et al., 2019). Since many secreted proteins, particularly glycoproteins, are folded and processed in the ER, we hypothesized that Dnj1 would play a role in capsule formation. When capsule was induced using low iron CIM, the *dnj1∆* deletion mutant was able to synthesize wild type-like capsule at 30°C (Figure 6A). However, during capsule induction with CIM at human body temperature (37°C), the *dnj1∆* mutant elaborated significantly smaller capsules than the wild type or complemented strains (Figure 6B). The *dnj1∆* mutant grown at 37°C also had increased aggregation suggesting a defect in cell separation, which may be related to the changes in cell wall architecture noted upon growth at 37°C (Figure 6C). We also observed that the capsules of the wild type and complemented strains were larger at 37°C than the capsules produced at 30°C (Figure 6C). Therefore, we propose that Dnj1 is required to support the increased secretory demand of producing a larger polysaccharide capsule at 37°C as this condition is more representative of the host condition.

**Figure 7:**
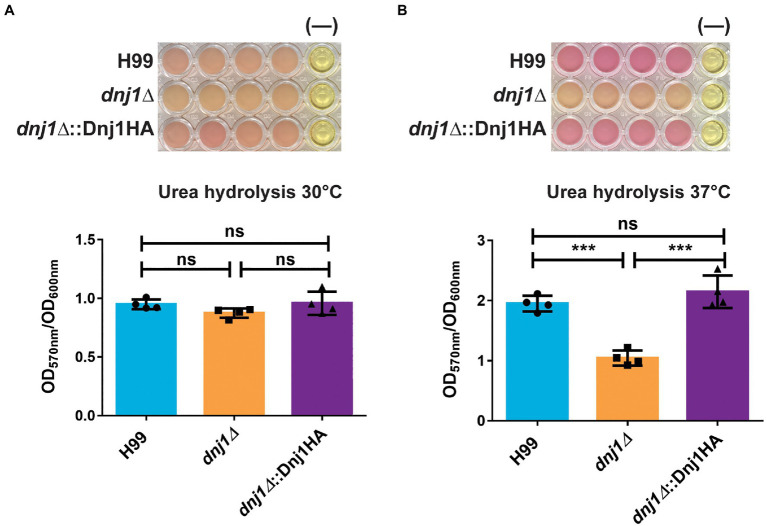
Dnj1 is required for robust extracellular urease activity in *C. neoformans*. Urease activity was assayed in minimal media containing 2% urea and phenol red as a pH indicator. The alkalinization caused by hydrolysis of urea to produce ammonia was measured by the change in the color of the media from yellow to pink and the OD_570nm_ of the supernatant. This quantification was normalized to OD_600nm_ to control for any differences in growth and a representative plate with a negative control (−) is shown for both temperatures assayed **(A)** 30 and **(B)** 37°C. Bars represent the mean OD_570nm_ of four biological replicates and the error bars indicate the SD. Significance was determined using a one way ANOVA with Tukey’s multiple comparison tests (^***^p<0.005).

The extracellular enzyme urease is another secreted factor which plays a role in the virulence of *C. neoformans* (Cox et al., 2000). In particular, secretion of urease is important for dissemination to the central nervous system as well as altering the host immune response to elicit a non-protective Th2 response (Olszewski et al., 2004; Osterholzer et al., 2009). We characterized urease activity using liquid minimal medium supplemented with urea and phenol red to assay the pH change when urea is hydrolyzed to generate ammonia. The activity of urease was lower in the mutant than the wild type upon growth at 37°C (Figures 7A,B). Taken together, the differences in capsule elaboration and urease activity between strains suggest that Dnj1 is required for robust delivery of virulence-associated factors to the cell surface at 37°C. These differences are consistent with the observed changes in cell wall composition and thickness in the *dnj1∆* mutant, although, we noted that there was no defect in the production of another major virulence factor, melanin, in the mutant (Supplementary Figure S4).

### Dnj1 Contributes to Virulence in a Mouse Model of Cryptococcosis

**Figure 8:**
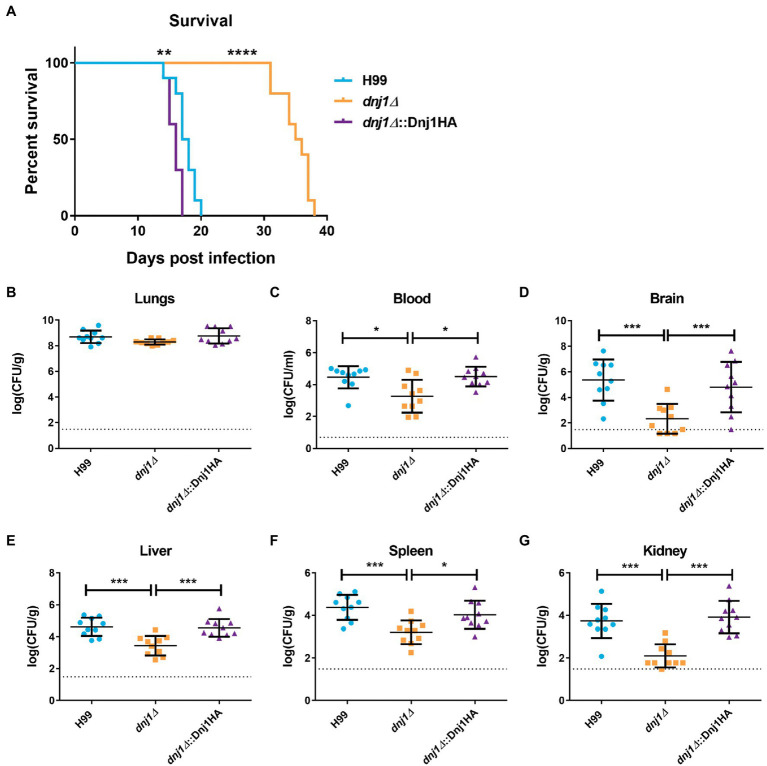
Dnj1 contributes to virulence and dissemination of *C. neoformans* in a mouse model of cryptococcosis. **(A)** Mice infected with the wild type (H99) and complemented strains (*dnj1Δ*::Dnj1HA) succumbed to infection between 14 and 20days, whereas mice infected with the *dnj1Δ* deletion mutant survived to between 31 and 38days post infection. Survival differences were determined using a log-rank test (^**^p<0.01 and ^****^p<0.001). **(B–G)** The fungal loads for mice infected with each strain were determined by measuring colony-forming units (CFUs) retrieved from the indicated homogenized tissues. Each dot represents the CFUs recovered from one mouse and the mean and SD are indicated for each group. The dashed line indicates the limit of detection for each organ based on the dilutions used in the experiment. Statistical significance of the differences observed were evaluated by Mann-Whitney U tests (^*^p<0.05 and ^***^p<0.005). Dashed horizontal lines indicate the limit of detection for the CFUs.

Both capsule and urease are secreted factors which influence virulence as well as the host immune response to *C. neoformans*. Since the *dnj1∆* deletion mutant had decreased expression of these factors at mammalian body temperature, we hypothesized that this strain would have reduced virulence. Therefore, we evaluated the requirement for *DNJ1* in virulence by employing a murine intranasal infection model of cryptococcosis and comparing disease progression in mice infected with the *dnj1∆* vs. the wild type and *dnj1∆*::Dnj1HA complemented strains. Mice infected with the wild type and complemented strains succumbed to the infection between 14 and 20days after intranasal inoculation (Figure 8A). In contrast, the mice infected with the *dnj1∆* mutant survived for significantly longer, succumbing to the infection between 31 and 38days post inoculation (Figure 8A). The fungal burden at the humane endpoint was also assessed and although there were no significant differences in the primary site of infection, the lung (Figure 8B), the mice infected with the *dnj1∆* deletion strain had significantly lower fungal burdens in the brain, i.e., the site most relevant for cryptococcal meningitis (Figure 8D). The mice infected with the *dnj1∆* mutant also had significantly lower fungal burdens in the blood as well as the systemic organs tested (liver, spleen, and kidney; Figures 8C,E–G). Taken together these results indicate that the *dnj1∆* mutant had decreased proliferation and, importantly, reduced dissemination compared to the wild type in a murine host.

**Figure 9:**
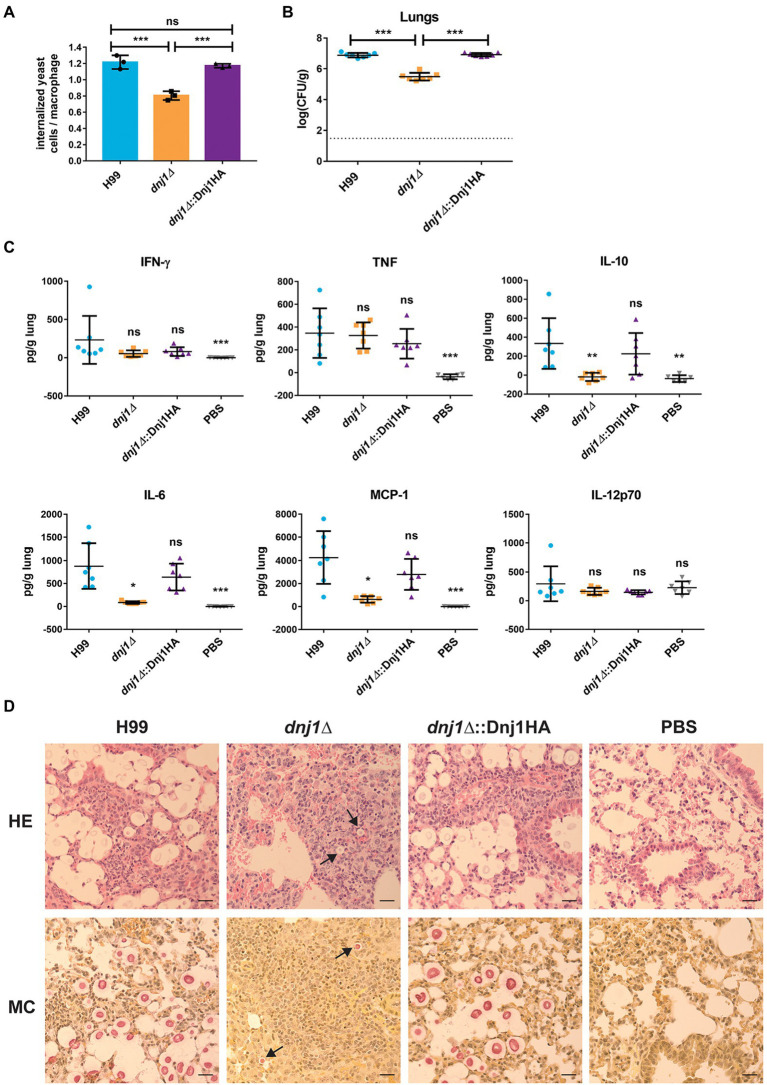
The attenuated virulence of the *dnj1∆* mutant may be attributed to decreased uptake by macrophages, an altered immune response, and slower proliferation early in a mouse model of cryptococcosis. **(A)** The proportion of internalized *C. neoformans* cells per macrophage for J774.A1 murine macrophages infected with the wild type (H99), *dnj1∆* mutant, and the *dnj1∆*::Dnj1HA complement. Significance was determined by a one-way ANOVA with Tukey’s multiple comparisons. **(B)** The fungal burdens in the lungs collected 6days post infection were also determined and statistical significance was determined by Mann-Whitney U tests. The dashed line indicates the limit of detection for CFU determination. **(C)** The cytokine profiles in the lungs of mice infected with wild type (H99), *dnj1∆* mutant, *dnj1∆*::Dnj1HA complement, or mock infected (PBS), were determined using a cytometric bead array mouse inflammation kit at 6days post infection. All significance shown was determined using one-way ANOVAs with Tukey’s multiple comparisons and the differences are relative to the wild type (H99) infection (ns, not significant; ^*^p<0.05, ^**^p<0.01 and ^***^p<0.005). **(D)** Histology micrographs of mouse lungs collected 6days after inoculation with each strain stained with hematoxylin and eosin (HE) or mucicarmine (MC). Arrows indicate *C. neoformans* in the *dnj1∆* mutant. Images are representative of lung histology from three mice. Scale bar=25μm.

To better understand the *in vivo* virulence defect, we examined the uptake of the mutant by murine macrophages and interrogated disease progression by examining an early point during infection. The *dnj1∆* mutant had decreased uptake by J774.A1 murine macrophages compared to the wild type or complemented strains (Figure 9A). This may explain the decrease in dissemination to the brain and systemic organs in the murine model of infection as *C. neoformans* is thought to be disseminated at least in part through transiting within the host, most notably across the blood-brain-barrier in macrophages (Charlier et al., 2009). To assess an early stage of infection, we employed the intranasal murine infection model to measure the fungal burden and inflammatory response in mouse lungs at 6days post inoculation. We observed that the fungal burden in the lungs of mice infected with the deletion mutant was significantly lower compared to mice infected with the wild type or complemented strains at 6days post inoculation (Figure 9B). At this time point, the inflammatory response in lungs collected from wild type, *dnj1∆*, *dnj1∆*::Dnj1HA, and mock (PBS) infected mice was evaluated using a mouse cytokine bead array. Mice infected with all strains of *C. neoformans* stimulated both interferon gamma (IFN-γ) and tumor necrosis factor (TNF) in the lungs above the levels measured in the PBS control; however, there were no significant differences between the infected mice (Figure 9C). Interleukin 6 (IL-6), interleukin 10 (IL-10), and monocyte chemoattractant protein 1 (MCP-1) were induced by infection of both the wild type and complemented strains; however, they were present at significantly lower concentrations in the lungs of mice infected with the *dnj1∆* mutant (Figure 9C). Finally, IL12-p70 was not stimulated by *C. neoformans* infection above the PBS control background levels (Figure 9C).

A histological analysis at day 6 also revealed differences in the lungs of mice infected with the *dnj1∆* mutant (Figure 9D). Notably, mice infected with the wild type and complemented strains had numerous *C. neoformans* cells in the tissue while, in contrast, the mice infected with the *dnj1∆* mutant had relatively few cells; these cells were significantly smaller in size than the fungal cells in the lungs of mice infected with the wild type or complement and no cells approaching titan cell size were visualized in mice infected with the *dnj1∆* mutant (Supplementary Figure S5). Mucicarmine staining was used to stain the capsule polysaccharide (pink) and mouse tissue (yellow). This approach revealed large, clear halos around the pink cells in the lung tissue of mice infected with the wild type and complement strains, whereas very small halos were observed around the cells in mice infected with the *dnj1∆* mutant. This observation suggests that the *dnj1∆* mutant formed smaller capsules *in vivo* compared to the wild type or complemented strains, and this is consistent with the observation of *in vitro* capsule induction at mammalian body temperature (Figure 6; Supplementary Figure S5). Hematoxylin and eosin (HE) staining which stains mouse cell nuclei purple and the cytoplasm pink also revealed that mouse lungs infected with each of the strains of *C. neoformans* displayed infiltration of immune cells. Specifically, there were more immune cells and fewer empty alveolar spaces compared to the mock (PBS) inoculated mice (Figure 9D). Overall, the data collected from an early time point of infection suggested that the proliferation of the *dnj1∆* mutant is delayed in lung tissue and that an immune response was elicited by the mutant but there was less stimulation of several cytokines compared to the wild type and complemented strains. The slower proliferation likely explains the observed attenuated virulence for the *dnj1∆* mutant, despite the finding that the strain eventually reached levels comparable to the wild type strain in lung tissue (Figure 8).

## Discussion

In this study, we established the contribution of the ER-localized co-chaperone, Dnj1, to the elaboration of virulence factors in the opportunistic fungal pathogen *C. neoformans*. Importantly, we showed that Dnj1 is not only required for thermotolerance at 39°C, but also for maintaining cell wall architecture as well as the expression and secretion of virulence factors at the human body temperature of 37°C. Subsequently, the deletion mutant lacking *DNJ1* was shown to have slower proliferation, attenuated virulence, and reduced dissemination to the brain in a mouse model of cryptococcosis. Additionally, this co-chaperone was also important for membrane homeostasis as demonstrated by the hypersensitivity of the *dnj1∆* mutant to azole antifungal drugs.

Dnj1 was previously described as a putative ortholog of the *S. cerevisiae* protein Jem1 in a study focused on karyogamy in *C. neoformans*; however, it was not found to play an important role in karyogamy or mating (Lee and Heitman, 2012). Here, we demonstrate that Dnj1 is distinct from Jem1 orthologs in the Saccharomycotina based on amino acid sequence comparisons, and in fact shares closer similarity to the other TPR containing JDPs. The ortholog of Dnj1 in *U. maydis* was recently shown to contribute to virulence through promoting ER homeostasis, filamentation, and secretion of effectors (Lo Presti et al., 2016). Similarly, we found that Dnj1 in *C. neoformans* was required for tolerance to ER stress as well as robust elaboration of virulence factors including production of capsule and accumulation of urease activity in the extracellular milieu. Importantly, we focused on the influence of Dnj1 at human body temperature because of the impact of *C. neoformans* as an opportunistic pathogen of immunocompromised people. This focus revealed a novel role for Dnj1 not only required for survival at elevated temperatures representative of clinically relevant fevers, but also for the elaboration of virulence factors at 37°C. This analysis also revealed a role for Dnj1 in maintaining the cell wall architecture at elevated temperature. The mutant lacking *DNJ1* was not hypersensitive to cell wall stress in *U. maydis*. This is consistent with our results, although, we did find increased cell wall chitin, chitosan, and overall thickness when cells were grown at 37°C. Our results also fit with previous findings that chitin deposition is increased in *S. cerevisiae* mutants lacking genes involved in ER stress and quality control pathways such as *CNE1* (Arias et al., 2011).

Connections between ER stress and thermotolerance have previously been established in *C. neoformans*. In particular, the ER chaperones Kar2 and Lhs1 are consistently upregulated in response to temperature upshift (Chow et al., 2007; Cheon et al., 2011; Yang et al., 2017). The upregulation of Kar2 is dynamic, reaching a maximum after approximately 1h of temperature upshift to 37°C and then returning to pre-stressed levels through Ccr4-mediated mRNA decay (Havel et al., 2011). This observation suggests that the ER chaperones are able to mitigate the stress induced by temperature upshift, adjust to the new folding capacity, and establish a new normal (Glazier and Panepinto, 2014). Based on our study, we hypothesize that Dnj1, as an ER co-chaperone, is required to facilitate the increased secretory demand of virulence factor production at 37°C. Furthermore, we propose that ER chaperones are coordinately required to maintain growth at modestly elevated temperatures. This idea is based on our observation that a mutant lacking both *DNJ1* and *CNE1* was unable to grow at 30°C, but room temperature was permissive for growth of this strain. Our study also revealed that temperature upshift may increase the burden on ER function and subsequently reduced ER homeostasis can impair the elaboration of virulence factors at elevated temperature.

Dnj1 contains seven tetratricopeptide repeat motifs. These motifs facilitate protein-protein interactions and are often involved in coordinating multiprotein complexes. In the context of chaperone networks, TPR proteins allow binding of multiple substrates or chaperones and can facilitate the processing of a misfolded protein from one chaperone to the next (Graham et al., 2019). For example, the closest human ortholog, DnaJC3, has been shown to bind ER lumenal substrates through its TPR domains (Petrova et al., 2008). It has also been shown to co-immunoprecipitate with newly synthesized secretory proteins and to promote maturation of proteins requiring post-translational modifications (Rutkowski et al., 2007). Similarly, we showed that Dnj1 in *C. neoformans* was present in the perinuclear ER, and was required for activity of secreted urease at 37°C. A secretion defect that influences the secretion of fungal factors may also explain the decrease of cytokine induction in the lungs of mice infected with the *dnj1∆* mutant compared to the wild type or complemented strains. Interestingly, we saw a decrease in both pro-inflammatory and anti-inflammatory cytokines. We hypothesize that this is likely due to a reduction in proliferation *in vivo*; however, it may also result from reduced secretion of immunomodulatory proteins from the *dnj1∆* mutant.

Dnj1 contributed to virulence in a murine model of infection as mice infected with the *dnj1∆* mutant survived more than 2weeks longer than those infected with the wild type or complemented strains. The lungs recovered from *dnj1∆* infected mice during an early stage of infection had fewer and smaller fungal cells compared to lungs infected with the wild type and complemented strains suggesting decreased proliferation early in infection. The *dnj1∆* mutant also had significantly impaired dissemination to the brain and other systemic organs. The attenuated virulence of this strain and its hypersensitivity to the azole drugs suggest that Dnj1 may be a promising target for antifungal drug development, potentially in combination therapy with existing azoles. Although, JDPs with ER function have not been targeted in fungal pathogens, JDPs are of considerable interest in other eukaryotic pathogens such as *Plasmodium falciparum* because of their roles in virulence (Külzer et al., 2010; Jha et al., 2017). Recently, an essential ER JDP in *P. falciparum*, *Pf*J2, was shown to have a druggable interaction with protein disulfide isomerases, which could be inhibited using a small molecule 16F16 (Cobb et al., 2021). There are also small molecule inhibitors capable of disrupting TPR mediated protein-protein interactions with Hsp90 (Vasko et al., 2010; Ardi et al., 2011; Smith and Gestwicki, 2012). Therefore, there is precedent in attempting to disrupt protein-protein interactions mediated by TPR domains as therapeutic strategies.

The JDP’s are emerging as a highly diverse family of proteins which support functions essential to maintaining organellar homeostasis and facilitating virulence in fungal pathogens (Lo Presti et al., 2016; Xie et al., 2017; Horianopoulos et al., 2020; Son et al., 2020). Here, we report on an ER co-chaperone, Dnj1, in *C. neoformans* which supports ER homeostasis and contributes to the production of virulence factors at human body temperature. Subsequently, Dnj1 was shown to impact the host-pathogen interaction through decreased induction of cytokines in the lung, attenuated virulence, and reduced dissemination to the brain. Dnj1 is one of several ER chaperones shown to play a role in virulence and there are several other uncharacterized ER chaperones and co-chaperones in *C. neoformans*, which may play distinct functions in fungal pathogenesis. Disruption of these chaperone networks could present a promising antifungal strategy to disturb ER homeostasis, potentiate azole drugs, and ultimately treat cryptococcosis.

## Data Availability Statement

The raw data supporting the conclusions of this article will be made available by the authors, without undue reservation.

## Ethics Statement

The animal study was reviewed and approved by UBC Animal Care Committee (ACC).

## Author Contributions

LH and JK were responsible for the experimental design, prepared the figures, and wrote the manuscript. LH, CL, GH, and MC conducted the experiments and analyzed the data. CL, GH, MC, and JK edited, revised, and approved the final manuscript. All authors contributed to the article and approved the submitted version.

## Funding

This work was supported by grants (MOP-13234 and PJT-166043) from the Canadian Institutes of Health Research (to JK) and a doctoral scholarship from the National Sciences and Engineering Research Council of Canada (to LH). Additional support was obtained from a Deutsche Forschungsgemeinschaft-International Research Training Group and an NSERC–CREATE Training Program (PRoTECT). JK is a Burroughs Wellcome Fund Scholar in Molecular Pathogenic Mycology, and a fellow of the CIFAR Program: Fungal Kingdom, Threats & Opportunities.

## Conflict of Interest

The authors declare that the research was conducted in the absence of any commercial or financial relationships that could be construed as a potential conflict of interest.

## Publisher’s Note

All claims expressed in this article are solely those of the authors and do not necessarily represent those of their affiliated organizations, or those of the publisher, the editors and the reviewers. Any product that may be evaluated in this article, or claim that may be made by its manufacturer, is not guaranteed or endorsed by the publisher.

## Footnotes

^1^ https://www.uniprot.org/

^2^ https://toolkit.tuebingen.mpg.de/tools/tprpred

^3^ https://github.com/mdbaron42/pyBoxshade
