# Supplementary material for: Dnj1 Promotes Virulence in Cryptococcus neoformans by Maintaining Robust Endoplasmic Reticulum Homeostasis Under Temperature Stress
Source: Front Microbiol. 2021 Sep 10;12:727039. doi: 10.3389/fmicb.2021.727039 (PMC8461255; doi:10.3389/fmicb.2021.727039)
Supplement: Supplementary file 1 [file Data_Sheet_1.PDF]

Supplementary Material

Supplementary Table S1. Strains used in the characterization of Dnj1. For each strain generated for this study, the resistance marker and the background strain which was transformed to construct it are indicated.

Strain	Genotype	Background
*dnj1*Δ	*dnj1::NEO*	H99
*dnj1*Δ::Dnj1HA	*Dnj1HA::NAT*	*dnj1*Δ
Dnj1-GFP	*Dnj1GFP::NAT*	*dnj1*Δ
*cne1*Δ	*cne1::NAT*	H99
*dnj1cne1*ΔΔ	*dnj1::NEO*<br>*cne1::NAT*	*dnj1*Δ

Supplementary Table S2. Primers and plasmids used in the characterization of Dnj1. The primer names and sequences used to generate the constructs used in this study are listed. The templates listed provide information on whether this part of the construct was amplified from H99 gDNA, Mrj1HA gDNA, or from one of the plasmids listed. The partner for each primer is indicated in the final column.

Primer name	Sequence 5'-3'	Plasmids and templates	Primer pair
Dnj1-1	CGGTGCTTGCTTGACCATAA	H99	Dnj1-2
Dnj1-2	AGCTCACATCCTCGCAGCGAACAGCGATTTATCCCGGC	H99	Dnj1-1
Dnj1-3	GCCGGGATAAATCGCTGTTCGCTGCGAGGATGTGAGCT	pJAF1	Dnj1-4
Dnj1-4	AGGCCCAACAGTACTAGTTCCGAAGAGATGTAGAAACTA	pJAF1	Dnj1-3
Dnj1-5	TAGTTTCTACATCTCTTCGGAACTAGTACTGTTGGGCCT	H99	Dnj1-6
Dnj1-6	ACGTTCGACAATTTGCTGGG	H99	Dnj1-5
Dnj1HA-1	CGGTGCTTGCTTGACCATAA	H99	Dnj1HA-2
Dnj1HA-2	GGGACATCGTAAGGGTAGTTCCACTGGAAGTGCATCTTC	H99	Dnj1HA-1
Dnj1HA-3	GATGCACTTCCAGTGGAACTACCCTTACGATGTCCCTGATTACG	Mrj1HA	Dnj1HA-4
Dnj1HA-4	GCTAGGCCCAACAGTAAGATGTAGAAACTAGCTTCCTGG	Mrj1HA	Dnj1HA-3
Dnj1HA-5	AGCTAGTTTCTACATCTTACTGTTGGGCCTAGCCGTG	H99	Dnj1HA-6
Dnj1HA-6	TGATAACCTTCGATGGCTCTCG	H99	Dnj1HA-5
Dnj1GFP-2	CTCCTCGCCCTTGCTCACGTTCCACTGGAAGTGCATCTTC	H99	Dnj1-1
Dnj1GFP-3	GCACTTCCAGTGGAACGTGAGCAAGGGCGAGGAG	pWH091	Dnj1GFP-4
Dnj1GFP-4	GAGCATGCATCTAGAGGAGATGGACCTGTTTCGTCTTTGC	pWH091	Dnj1GFP-3
Dnj1GFP-5	CGAAACAGGTCCATCTCCTCTAGATGCATGCTCGAGC	Dnj1HA	Dnj1HA-6
Cne1-1	CGATGTCGGTACTGGCTTGG	H99	Cne1-2
Cne1-2	GCCGTCGTTTTACAACACGGATGGGATGAATGGAAGACG	H99	Cne1-1
Cne1-3	CTTCCATTCATCCCATCCGTGTTGTAAAACGACGGCCAGT	pCH233	Cne1-4
Cne1-4	CTCAACTAAACCATTCGAGATGTAGAAACTAGCTTCCTGG	pCH233	Cne1-3
Cne1-5	AAGCTAGTTTCTACATCTCGAATGGTTTAGTTGAGCTGCC	H99	Cne1-6
Cne1-6	CTTGCTTGACGCTACCTGTGC	H99	Cne1-5

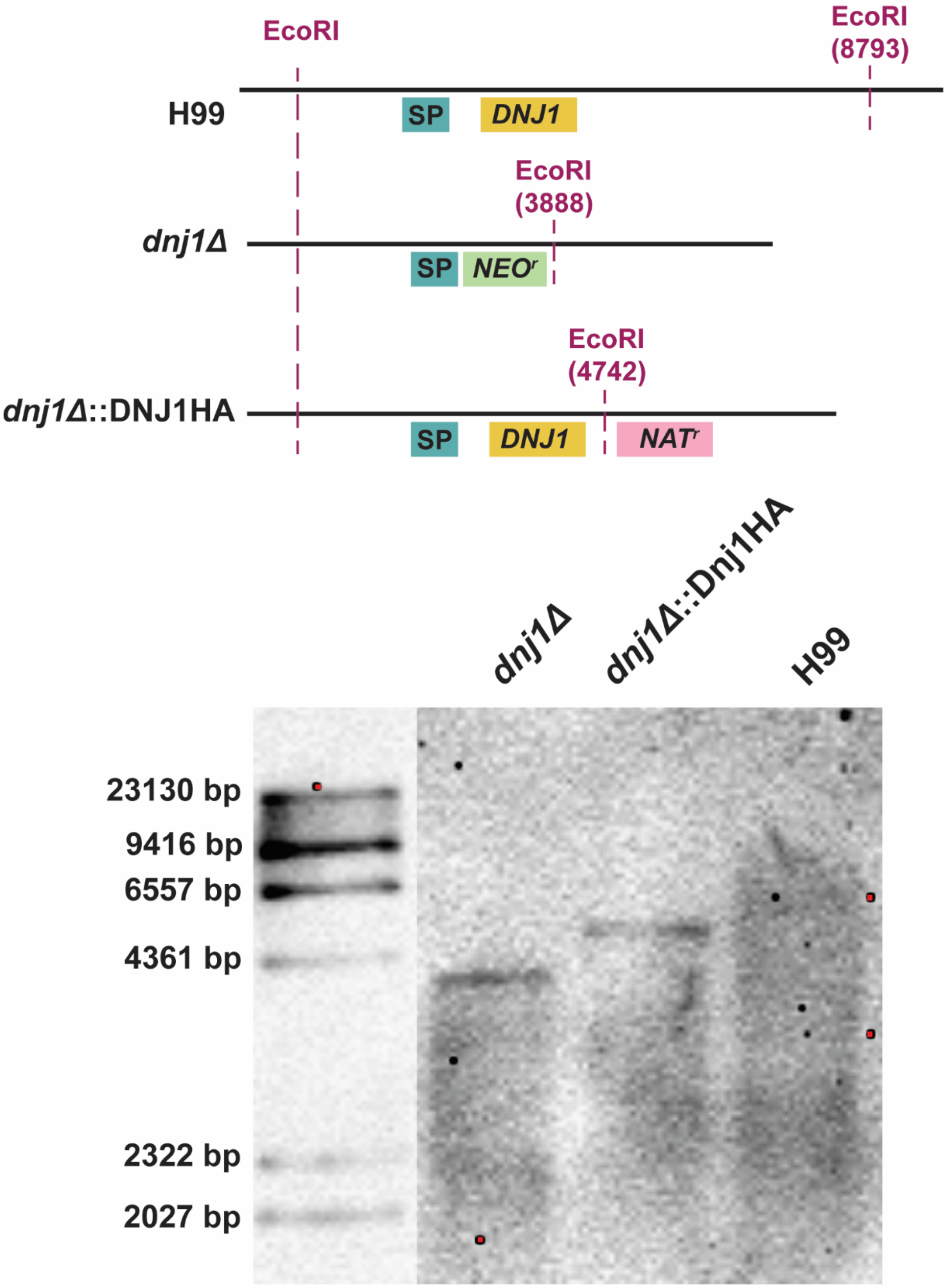

Supplementary Figure S1. Southern hybridization confirmation of the genotypes of the *dnj1Δ* and *dnj1Δ*::Dnj1HA strains. DNA from the indicated strains was extracted, digested with EcoRI at the indicated dashed lines, and genomic hybridization was performed using a DIG-labelled DNA probe (SP). The probe was used to detect fragments of 3888 bp in the deletion mutant, 4742 bp in the complemented strain and 8793 bp in the wild type (H99). A DIG labelled DNA ladder is also shown, however since it was oversaturated at the point that the hybridization bands were well visualized, this lane has been provided from an earlier time point in a sequential exposure of the blot. Note that the

genotypes of multiple transformants for the *dnj1Δ* construct were also confirmed by PCR in comparison with the wild type strain.

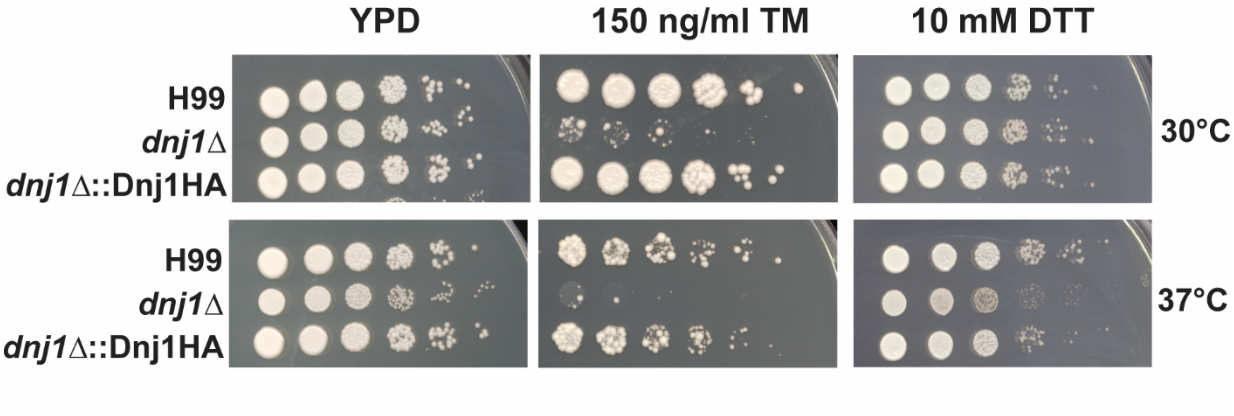

Supplementary Figure S2. ***DNJ1*** **is required for growth in the presence of ER stress.** The *dnj1*Δ deletion mutant was hypersensitive to tunicamycin compared to the wild type (H99) and complemented (*dnj1*Δ::Dnj1HA) strains. The *dnj1*Δ mutant was also mildly hypersensitive to the reducing agent dithiothreitol (DTT) when grown with the added stress of elevated temperature (37°C).

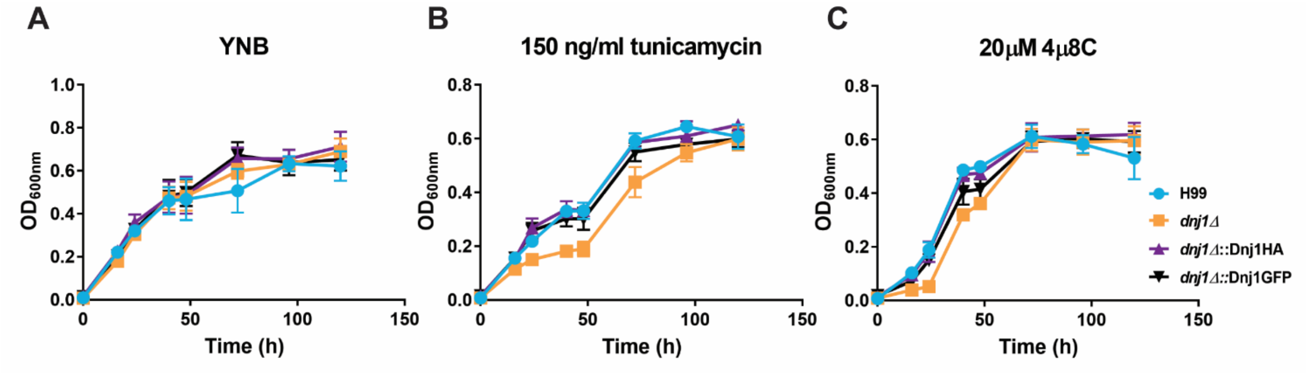

Supplementary Figure S3. A Dnj1GFP fusion protein complements the growth defects of the *dnj1*Δ deletion mutant in ER stress. Growth curves in **(A)** control media (liquid YNB + 0.5% dextrose) and YNB supplemented with ER inhibitors revealed that the hypersensitivity of the deletion mutant *dnj1*Δ to **(B)** 150 ng/ml tunicamycin (TM) and **(C)** 20 µM 4µ8C was restored to wild type (H99) levels in the GFP-tagged, complemented strain (*dnj1*Δ::Dnj1GFP). The error bars represent the standard deviation of 4 biological replicates.

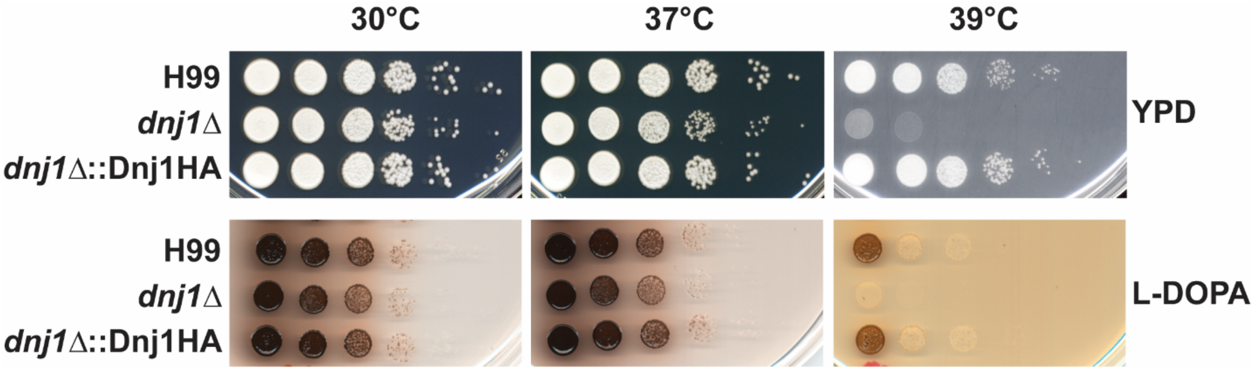

Supplementary Figure S4. Melanin production in mutants lacking *DNJ1*. Spot assays of the wild type (H99), *dnj1*Δ mutant, and complemented strains (*dnj1*Δ::Dnj1HA) serially diluted and plated on YPD and L-DOPA agar to assess melanin formation, with incubation at 30°C, 37°C, and 39°C as indicated.

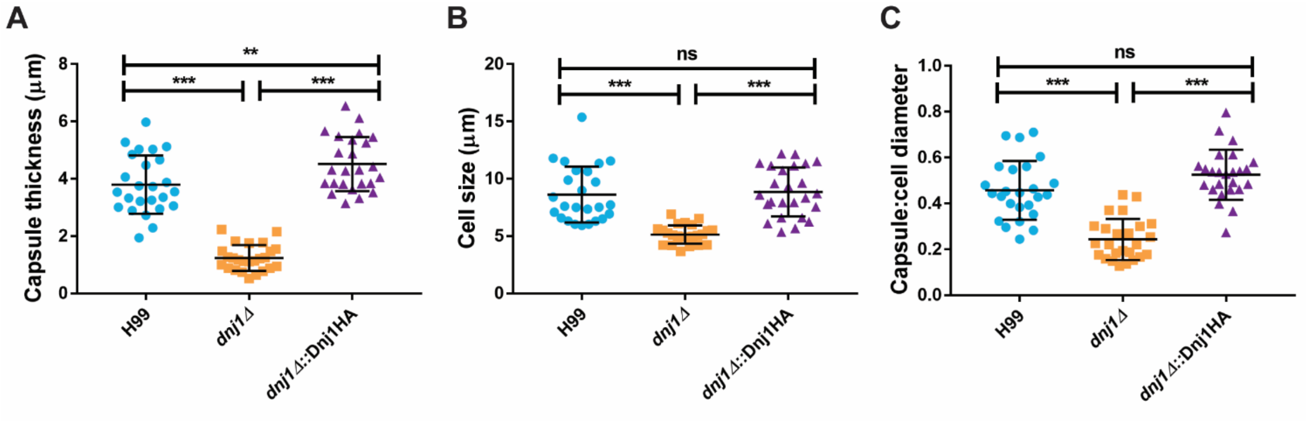

Supplementary Figure S5. Capsule thickness and cell size are decreased *in vivo* in the *dnj1*Δ mutant. After quantification of cell and capsule size for 25 cells from mucicarmine staining of histological images of mouse lungs infected with *C. neoformans,* the **(A)** capsule thickness and **(B)** cell size were significantly smaller in mice infected with the *dnj1*Δ deletion mutant. **(C)** The capsule thickness to cell size ratio was also significantly smaller for the *dnj1*Δ deletion mutant suggesting that the smaller capsules are not only a function of cell size differences.
